# Dr. Mordecai Price

**Published:** 1904-12

**Authors:** 


Dr. Mordecai Price, of Philadelphia, died suddenly at his home in that city, October 29, 1904, aged 60 years. A more extended notice of this eminent surgeon will appear in a subsequent issue of the Journal.

